# Relationships among physical activity, sleep duration, diet, and academic achievement in a sample of adolescents

**DOI:** 10.1016/j.pmedr.2018.08.014

**Published:** 2018-08-28

**Authors:** Ryan D. Burns, You Fu, Timothy A. Brusseau, Kristen Clements-Nolle, Wei Yang

**Affiliations:** a Department of Health, Kinesiology, and Recreation, University of Utah, Salt Lake City, UT, USA; b School of Community Health Sciences, University of Nevada Reno, Reno, Nevada, USA

**Keywords:** Adolescents, Academic performance, Diet, Physical activity, Sleep

## Abstract

The purpose of this study was to examine the relationships among physical activity, sleep duration, diet, and academic achievement in a sample of adolescents from the US state of Nevada. A two-stage cluster random sampling method was used to recruit Nevadan adolescents (N = 4625). The 2015 Youth Risk Behavior Survey was administered to students within public, private, and charter schools. Weighted multilevel generalized linear mixed effects models were employed to examine the relationships among physical activity, sleep duration, diet, and academic achievement. Additional analyses were run to examine the relationship between meeting multiple health behavior criteria with academic achievement. Data were collected in the US state of Nevada in 2015 and analyzed in the US state of Nevada in 2018. Adolescents who participated in at least of 60 min of physical activity per day had significantly higher odds of achieving mostly A's and B's (adjusted OR = 1.18; 95% C.I.: 1.02, 1.38; p = 0.029). Additionally, adolescents who consumed salad weekly (adjusted OR = 1.24; 95% C.I.: 1.06, 1.46; p = 0.007) and who consumed breakfast everyday (adjusted OR = 1.72; 95% C.I.: 1.48, 2.00; p < 0.001) had higher odds of achieving mostly A's and B's. Finally, adolescents who reported meeting 3 or more health behavior criteria had significantly higher odds of achieving mostly A's and B's compared to adolescents meeting only 0–2 health behaviors (adjusted OR = 1.66; 95% C.I.: 1.44, 1.92; p < 0.001). Self-reported physical activity, specific dietary behaviors, and meeting multiple health behavior criteria significantly related to academic achievement in adolescents.

## 1 Introduction

Higher levels of physical activity has numerous benefits for adolescents including improved emotional well-being and decreased risk for cardio-metabolic disease (Ekelund et al., 2012; Fromel et al., 2017). One area that has been of increasing focus in research is the link between physical activity and cognitive functioning (Gomez-Pinilla & Hillman, 2013). This relationship is especially important to school-aged adolescents because improved cognitive functioning may lead to better classroom behavior and academic performance (Khan & Hillman, 2014; Pellicer-Chenoll et al., 2015). Given the link between physical activity and cognitive functioning, school-based interventions have been derived for the purposes of improving academic performance in youth by providing additional, expanded, or enhanced physical activity opportunities during the school day (e.g., physical education, recess, classroom) (Mahar, 2011; Burns et al., 2016; Stylianou et al., 2016).

It is becoming clear that physical activity and improved health-related fitness levels may lead to improved cognitive functioning and academic performance in adolescents. However, physical activity's independent relationship with academic performance considering other modifiable health behaviors is unclear within the adolescent population. Sleep is a health behavior that is also gaining attention in pediatric health behavior research (Cappuccio et al., 2008). Poor sleep duration and quality has been linked to increased risk behaviors and poorer academic performance in adolescents (O'Brien & Mindell, 2005; Hysing et al., 2016). Poor sleep behaviors, if sustained over time, may affect cardio-metabolic risk and even increase risk for depression and anxiety disorders (Quist et al., 2016; Ojio et al., 2016). It is suggested that adolescents should get at least 8 h of sleep per night (Hirshkowitz et al., 2015). What is unclear is whether physical activity, sleep duration and other health behaviors such as diet, when considered together, have a significant independent relationship with adolescent academic performance. To the authors' knowledge, no study to date has explored the independent relationships among physical activity, sleep duration, diet, and academic achievement in a state sample of US adolescents and if meeting multiple health behavior criteria relates to better academic performance. Therefore, the purpose of this study was to examine the relationships among physical activity, sleep duration, diet, and academic achievement in a sample of Nevadan adolescents. Given the link between health behaviors and cognitive functioning, it was hypothesized that physical activity, diet, and sleep behaviors would have significant independent relationships with academic performance.

## 2 Methods

### 2.1 Participants

The 2015 Nevada YRBS sampling plan was designed to ensure that every eligible student had an equal chance of selection. A two-stage (region and classroom) cluster sample design was used to produce a representative sample of students in grades 9–12 (all Nevada regular public, charter, and alternative high schools). In order to provide regional data that represent all counties in the state, the first sampling stage grouped all 17 school districts into 7 regions that generally reflect the substance abuse prevention coalition structure. The coalition structure is a prevention initiative titled “Nevada Statewide Coalition Partnership”. In the second sampling stage, classes were selected within schools with the probability based on a required margin of error in the sample size for each region. Intact classes of either all second period or all required English courses were randomly selected from each school. All collected data were weighted at the state level and regional level based on the sex, race/ethnicity, and grade level of students in each region. The final sample included 5108 adolescents from 97 schools with an overall response rate of 65% (Clements-Nolle et al., 2018). This study was approved by the University Institutional Review Board and when required the local school district Institutional Review Boards (Clements-Nolle et al., 2018).

## 3 Measures

The Youth Risk Behavior Survey (YRBS) was designed to monitor health-related behaviors among high-school students. Data from the 2015 Nevada YRBS were used in the current study (Clements-Nolle et al., 2018). Standardized YRBS variables were used to assess current physical activity “During the past 7 days, on how many days were you physical activity for a total of at least 60 minutes per day?”, sleep “On an average school night, how many hours of sleep do you get?”, and academic achievement “During the past 12 months, how would you describe your grades in school?”. Diet variables included eating fruit at least once per week, eating salad at least once per week, eating vegetables at least once per week, and having breakfast every day.

Data Processing.

Of the 5108 Nevadan adolescents who completed the YRBS, 4625 had answered all physical activity, sleep duration, diet, and academic performance questions. Because approximately 9.5% of the observations were missing, it was determined that bias was less likely compared to if there was a higher prevalence of missing data (Knol et al., 2010). Predictor and outcome variables were dichotomized to distinguish adolescents who reported a behavior from those who did not report any health behavior at all and also to distinguish those adolescents who met a health behavior guideline from those adolescents who did not (0 = not meeting, 1 = meeting). The outcome variable was maintaining grades that were mostly A's or mostly B's over the past year. The primary predictors were participating in at least 60 min of physical activity per day during the past week, sleeping for at least 8 h per day over the past week and meeting criteria for eating fruit, salad (at least once per week), vegetables (at least once per week), and breakfast (everyday). The referents for the aforementioned variables were not meeting the communicated criteria. A multicomponent health behavior variable was then derived summing the met health criteria and then dichotomizing the variable into adolescents who met 0–2 health behavior criteria and those who met 3 or more health behavior criteria.

### 3.1 Statistical analysis

Differences between sexes on all observed variables on the continuous and categorical measurement scales were analyzed using independent *t*-tests and weighted chi-square tests, respectively. To examine the associations among physical activity, sleep duration, diet, and academic achievement, weighted multi-level generalized linear mixed effects models with a logit link were employed. Sampling weights were calculated from region data. Random effects were employed at the region and classroom level to adjust for clustering within the data structure. Unadjusted and adjusted odds ratios were calculated with corresponding 95% Confidence Intervals. The adjusted model included the covariates of sex, age, age- and sex-specific BMI percentiles, and race/ethnicity. An additional weighted multi-level model was employed examining the effect of meeting at least 3 or more health behaviors on academic achievement. All non-linear categorical analyses were weighted using STATA's “survey” command. Alpha level was set at p ≤ 0.05 and analyses were conducted using STATA v15.0 statistical software package (College Station, Texas, USA).

## 4 Results

Descriptive data are communicated in Table 1. Boys had a higher mean BMI percentile compared to girls (mean difference = 4.5%, p < 0.001) and reported a greater proportion of eating breakfast everyday compared to girls (p < 0.001). A greater proportion of boys achieved 60 min of physical activity per day (p < 0.001) and who achieved 8 h of sleep or more (p < 0.001). However, a greater proportion of girls reported weekly consumption of fruit (p < 0.001), salad (p < 0.001), and vegetables (p < 0.001), in addition to a greater proportion achieving mostly A's and B's compared to boys (p < 0.001). Unadjusted and adjusted odds ratios from the generalized linear mixed effects models are reported in Table 2. In the adjusted model, adolescents who participated in at least 60 min of physical activity per day had significantly higher odds of achieving mostly A's or B's (*p* = 0.029). Additionally, adolescents who weekly consumed salad (adjusted OR = 1.24; 95% C.I.: 1.06, 1.46; p = 0.007) and who consumed breakfast everyday (adjusted OR = 1.72; 95% C.I.: 1.48, 2.00; p < 0.001) had higher odds of achieving mostly A's and B's. An additional analysis was conducted examining the influence of meeting multiple health behaviors. Adolescents who reported achieving 3 or more health behaviors had significantly higher odds of achieving mostly A's and B's compared to adolescents meeting only 0–2 health behaviors (adjusted OR = 1.66; 95% C.I.: 1.44, 1.92; p < 0.001). This relationship was independent of sex, age, sex and age-adjusted BMI percentile, and race/ethnicity.

**Table 1:** Descriptive data for the total sample and within sex groups.

		Total sample (N = 4625)	Girls (n = 2544)	Boys (n = 2081)
Predictors	Meeting 60 min per day of PA	49%	42.2%	**57.2%**
	8 or more hours of sleep per day	22.5%	19.2%	**25.2%**
	Consume Fruit	89.1%	**91.8%**	86.5%
	Consume Salad	61.7%	**66.0%**	57.3%
	Consume Vegetables	81.3%	**83.9%**	78.6%
	Consume Breakfast	34.6%	31.7%	**37.5%**
Outcome	Mostly A's or B's	70.2%	**74.6%**	65.9%
Covariates	14 years old	12.0%	12.5%	11.3%
	15 years old	27.5%	27.5%	27.6%
	16 years old	26.5%	26.6%	26.4%
	17 years old	23.0%	23.1%	23.0%
	18 years old	11.0%	10.3%	11.7%
	BMI Percentile	61.2% ± 29.2%	59.0% ± 29.5%	**63.5% ±** **28.8%**
	American Indian/Alaskan Native	2.1%	1.8%	2.4%
	Asian	5.2%	4.7%	5.6%
	African American	5.5%	5.0%	5.9%
	Pacific Islander	1.8%	1.5%	2.2%
	White	39.2%	38.7%	39.8%
	Hispanic/Latino	40.6%	42.2%	39.1%
	Other/Multiple Race/Ethnicity	5.6%	6.1%	5.0%

Note: PA stands for physical activity; BMI stands for Body Mass Index; bold denotes statistical differences between sexes, p ≤ 0.05; Data collected in the US state of Nevada in 2015; Data analyzed in the US state of Nevada in 2018.

**Table 2:** Unadjusted and adjusted odds ratios from generalized linear mixed effects models relating physical activity, sleep duration, and diet with academic achievement.

	Unadjusted model		Adjusted model	
	OR	95% C.I.	OR	95% C.I.
Meeting 60 min per day of PA	1.11	0.96, 1.27	**1.18**	1.02, 1.38
8 or more hours of sleep per day	**1.18**	1.01, 1.39	1.12	0.94, 1.34
Consume fruit	1.17	0.92, 1.50	1.23	0.95, 1.60
Consume salad	**1.30**	1.12, 1.51	**1.24**	1.06, 1.46
Consume Vegetables	1.06	0.88, 1.29	1.05	0.85, 1.29
Consume breakfast	**1.75**	1.51, 2.03	**1.72**	1.48, 2.00
Sex			**1.80**	1.54, 2.10
15 years old			0.82	0.64, 1.05
16 years old			**0.67**	0.50, 0.91
17 years old			**0.68**	0.50, 0.92
18 years old			0.78	0.54, 1.13
BMI percentile			0.99	0.99, 1.00
Asian			**5.41**	2.80, 10.48
African American			**1.80**	1.05, 3.10
Pacific Islander			**3.41**	1.62, 7.16
White			**2.73**	1.66, 4.50
Hispanic/Latino			1.39	0.86, 2.27
Other/Multiple race/Ethnicity			**1.90**	1.07, 3.36

Note: All presented data based on the weighted estimation sample; OR stands for Odds Ratio; PA stands for physical activity; BMI stands for Body Mass Index; outcome variable is maintaining mostly A's or B's over the past academic year; referent for physical activity is not meeting 60 min per day; referent for sleep duration is not sleeping at least 8 h per day; referents for diet behavior is not displaying the respective behavior; referent for sex is males; referent for age is 14 years old; referent for ethnicity is American Indian/Alaskan Native; bold denotes statistical significance, p ≤ 0.05; Data collected in the US state of Nevada in 2015; Data analyzed in the US state of Nevada in 2018.

## 5 Discussion

The purpose of this study was to examine the relationships among physical activity, sleep duration, diet, and academic performance in a sample of adolescents from Nevada. After controlling for sex, age, BMI percentile, and race/ethnicity, daily physical activity was associated with academic performance. Consuming salads weekly and having breakfast everyday also were associated with academic achievement. Additional analyses revealed that adolescents who achieved 3 or more health behavior criteria had significantly higher odds of maintaining mostly A's and B's over the past academic year compared to adolescents only reporting 0–2 health behavior criteria.

It has been suggested that various adolescent health behaviors may work in consort to yield effects on various outcomes such as academic performance (Bradley & Greene, 2013; Martínez-Gómez et al., 2012). These effects may be additive when considered together (Bradley & Greene, 2013; Martínez-Gómez et al., 2012). However, testing their independent effects when considered together on academic achievement are unclear within the US adolescent pediatric population. Martínez-Gómez et al. (2012) found that physical activity related to academic performance in Spanish adolescent girls, but not in boys. Martínez-Gómez et al. (2012) also found that Spanish adolescent girls who met recommendations for multiple health behaviors had better academic performance than girls who met recommendations on 0–1 health behaviors. Ickovics et al. (2014) found that US middle school students that met standards on multiple health behaviors were more likely to meet goals for standardized tests. Past studies have established that multiple health behavior components may have greater beneficial effect on academic performance, but the relative contribution of each health behavior is unclear. Faught et al. (2017) found no associations between physical activity, assessed using the PAC-C, and meeting expectations in mathematics in Canadian elementary school children, but did find significant associations with sedentary screen time and meeting expectations in mathematics and writing. The differences found in the current study's results concerning physical activity from previous studies could be the use of an older sample of adolescents, and how physical activity is defined, assessed, stratified, and controlled for with specific covariates. The current study suggests that physical activity has a relationship with academic performance controlling for pertinent confounders such as sleep duration, BMI, and diet in a state sample of adolescents. The current study also supports past research stating that meeting criteria for multiple health behaviors has a greater relationship with academic achievement than meeting criteria on zero or a few health behaviors. These findings have important implications when deriving school and community-based interventions.

There are limitations to this study that must be considered. First, the participants were a sample of Nevadan adolescents; therefore, the results cannot be generalized to other populations of youth. Second, the study design was cross-sectional; therefore, no causal inferences can be made. Despite our hypotheses driven by the potential causal link between health behavior and academic achievement, it is possible that academic achievement has a causal effect on the observed health behaviors; that is, higher levels of academic achievement lead to improved physical activity, sleep, and diet. These potential links should be explored with additional research. Third, data were collected using self-report methods, which raises the question of and social desirability bias. Given that approximately 35% of the recruited sample were non-respondents, there was potential for selection bias. Therefore, it is possible that adolescents who display poor health behaviors did not respond to the questionnaire, which may have distorted the findings. Additionally, physical activity was assessed using one question without clear definition or an assessment of specific physical activity intensity. The construct validity of physical activity would have been stronger using a more comprehensive self-report instrument. Fourth, there may be residual confounding as not all potential confounding variables were assessed in the YRBS. This may especially be true for the potential confounders of socio-economic status and parental education. Unfortunately, using questionnaires designed for adolescent response, socio-economic status and parental education information may be difficult to obtain. Finally, mediating relationships among physical activity, sleep, and diet on the academic achievement outcome were not assessed; this should be a priority for future research.

## 6 Conclusion

This study supports the link between daily physical activity, diet, and academic achievement in a sample of Nevadan adolescents. This relationship is independent of sex, age, BMI percentile, and race/ethnicity. Additionally, meeting criteria on multiple health behaviors seems to have a relationship with better academic achievement compared to meeting zero to few health behavior criteria. The results of this study can be used to support and inform school and community-based interventions with aims to employ multicomponent health behavior modification to improve academic achievement in adolescents.

## Conflicts of interest

No conflicts of interest were reported by the authors of this paper.
